# Rare Association of Extended Total Colonic Aganglionosis and Intestinal Malrotation

**DOI:** 10.21699/jns.v5i4.468

**Published:** 2016-10-10

**Authors:** Iulia Stratulat-Chiriac, Danielle Mc Laughlin, Brice Antao

**Affiliations:** Department of Paediatric Surgery, Our Lady's Children's Hospital Crumlin, Dublin, Ireland

**Keywords:** Total colonic aganglionosis, Hirschsprung's disease, Malrotation, Intestinal obstruction, Neonate

## Abstract

Total colonic aganglionosis occurring together with malrotation is a rare occurrence and may pose diagnostic and management dilemmas for the paediatric surgeon. We report a case of a neonate that presented with extended total colonic aganglionosis and malrotation, along with a spectrum of central nervous system and renal abnormalities. The clinical and radiological features and potential diagnostic and management pitfalls are discussed along with a literature review of this exceptionally infrequent association.

## INTRODUCTION

Hirschsprung's Disease (HD) and malrotation can arise together in a small proportion of cases [1]. Symptomatic malrotation has been reported to occur in 1 in 6000 live births. However, malrotation may be an incidental finding in up to 1 in 500 live births during surgery or radiological investigations [2]. Malrotation is frequently diagnosed in association with other congenital pathologies of the bowel, diaphragm and abdominal wall [2]. The incidence of HD is 1 in 5000 live births. Total colonic aganglionosis (TCA) accounts for approximately 10% of cases of HD, and some of these patients have aganglionosis extending into the small bowel. Patients with extended aganglionosis encompass a rare and severe phenotype and present a difficult surgical challenge with higher rates of morbidity and mortality [3]. 

We recently managed a neonate presenting with bilious vomiting for whom the diagnosis of malrotation was made radiologically and surgically. This ultimately led to a significant delay in definitive diagnosis and appropriate surgery for the true obstructive bowel pathology. This case illustrates an important lesson in the surgical management of neonatal bowel obstruction.

## CASE REPORT

A female neonate was born at 36+4/40 weeks gestation with a birth weight of 2.76 kg by caesarean section for breech presentation to a 15 year old primigravida mother. Antenatal anomaly scanning at 25 weeks gestation detected a cystic abnormality of the left kidney. The patient had passed a small amount of meconium within the first 24 hours of life. She presented to our centre on day 4 of life with feed intolerance and bilious vomiting. Clinical examination revealed moderate abdominal distension but was otherwise unremarkable. An urgent upper gastrointestinal contrast study (UGI) was diagnostic for malrotation (Fig.1A). The patient subsequently underwent emergency laparotomy which confirmed malrotation without volvulus. The bowel was otherwise normal in appearance and a Ladd procedure was performed. 

**Figure:**
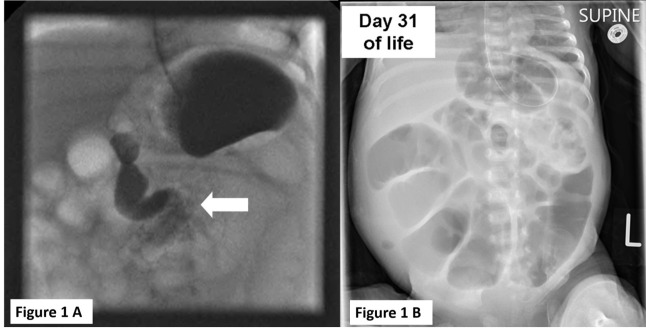
Figure 1: (A) Upper gastrointestinal contrast study performed on Day 4 of life showing D-J flexure position low and at the midline (arrow). (B) X-ray of the Abdomen showing persistent dilated loops 3 weeks post-Ladd procedure.

The post-operative course was complicated by failure to establish feeds, total parenteral nutrition (TPN) dependence and ongoing large volume bilious aspirates from the nasogastric tube. Associated anomaly workup during this period revealed bilateral optic nerve hypoplasia, bilateral peri-sylvianpolymicrogyria, absent septum pellucidum and septo-optic dysplasia on MRI brain and polycystic left kidney with ectopic left ureter on renal ultrasonography. Parents were non-consanguineous and genetics workup was normal. 

Ongoing obstructive symptoms (Fig.1B) beyond the 3rd postoperative week including feculent vomits and failure of spontaneous bowel opening prompted further evaluation for alternative pathology including a rectal suction biopsy which revealed an absence of ganglion cells on calretinin staining, indicating a diagnosis of HD. On Day 40 of life a second laparotomy was undertaken. Following adhesiolysis (from the previous Ladd procedure) a calibre change at the mid-small bowel was noted. Levelling frozen section biopsies confirmed total colonic aganglionosis extending into the small bowel to 50cms from the ileocecal valve. Resection of the aganglionic small bowel and subtotal colectomy to the level of the distal sigmoid was performed and an end ileostomy was formed at 140cm from the Duodeno-Jejunal (D-J) flexure.

The subsequent postoperative period was complicated by wound dehiscence, sepsis and stoma necrosis. On Day 47 of life, the patient underwent refashioning of her end ileostomy and wound closure. Recovery was further protracted as a result of high stoma losses. She was gradually weaned off TPN and eventually established on full enteral feeds by Day 70 of life. She is currently under regular surgical and medical review while being cared for at home and will be managed with an ileostomy for the foreseeable future.

## DISCUSSION

The present case was a challenging scenario for us where a diagnosis of malrotation had obscured the true causative pathology as HD. The obstructive symptoms at presentation were due to TCA and malrotation was probably an incidental finding. But in the setting of bilious vomiting with evidence of malrotation on UGI, laparotomy was mandatory. 

While many cases of bilious vomiting are ultimately not due to obstructive bowel pathology and a finding of malrotation may be incidental, the surgeon must be conscious of the association of malrotation with other congenital gastro-intestinal anomalies to avoid misdiagnosis. Confirmation of malrotation but exclusion of volvulus at laparotomy compels a thorough inspection of the entire bowel to detect atresia or transition zone in cases of HD to allow appropriate intervention at the same sitting [2]. In our case the extended small bowel aganglionosis, which is a rare phenomenon, was not apparent at laparotomy due to failure of the bowel to distend. The passage of meconium within 24 hours of birth and the intra-operative findings assuaged the suspicion of other intestinal anomalies and delayed the diagnosis of HD.

Prolonged intestinal obstructive symptoms following Ladd procedure without volvulus and failure of spontaneous bowel movement, prompted us to investigate for alternative diagnoses and perform a rectal biopsy. At subsequent laparotomy this directed our management towards levelling biopsies where we may have otherwise just undertaken an adhesiolysis. Failure to identify the need for a rectal biopsy would have further protracted the diagnosis with increased morbidity and warranted further operative intervention. 

Importantly, of 15 cases of TCA with associated malrotation reported in the literature, patients had both asymptomatic malrotation incidentally diagnosed and malrotation with volvulus presenting as an emergency [4-8]. Even when evidence of other congenital bowel anomalies is present, it is necessary to remain vigilant to the evolving clinical picture, as malrotation remains a dangerous entity in the setting of HD.

In conclusion, the following learning points are proposed: In neonatal bilious vomiting or bowel obstruction one must keep in mind the association of other anomalies such as HD with malrotation; In cases of HD and especially for TCA occurring in association with malrotation, the clinical examination as well as the radiological and surgical findings can be non-specific; A high index of suspicion is necessary for prompt diagnosis and early management of both malrotation and HD; Persistent intestinal obstructive symptoms following a Ladd procedure, particularly in the absence of volvulus, is a red flag and warrants an early rectal suction biopsy, prior to consideration of a subsequent laparotomy.

## Footnotes

**Source of Support:** Nil

**Conflict of Interest:** Nil
